# Gynecologic assessment of 19 adult females with cartilage-hair hypoplasia – high rate of HPV positivity

**DOI:** 10.1186/s13023-018-0945-9

**Published:** 2018-11-16

**Authors:** Elina Holopainen, Svetlana Vakkilainen, Outi Mäkitie

**Affiliations:** 1 Women’s Hospital, HUS Helsinki University Hospital and University of Helsinki, P.O. Box 140, FIN-00029 HUS Helsinki, Finland; 2 Children’s Hospital, Pediatric Research Center, HUS Helsinki University Hospital and University of Helsinki, Helsinki, Finland; 3 Institute of Genetics, Folkhälsan Research Center, Helsinki, Finland; 4 Department of Molecular Medicine and Surgery, Karolinska Institutet, Stockholm, Sweden; 5 Department of Clinical Genetics, Karolinska University Hospital, Stockholm, Sweden

**Keywords:** Skeletal dysplasia, Contraception, Human papillomavirus, CHH, *RMRP*

## Abstract

**Background:**

Patients with cartilage-hair hypoplasia (CHH), a rare metaphyseal chondrodysplasia, manifest severe growth failure, variable immunodeficiency and increased risk of malignancies. The impact of CHH on gynecologic and reproductive health is unknown. Vulnerability to genital infections may predispose CHH patients to prolonged human papillomavirus (HPV) infections potentially leading to cervical, vaginal and vulvar cancer.

**Methods:**

We carried out gynecologic evaluation, pelvic ultrasound and laboratory assessment in 19 women with genetically confirmed CHH. All patients were clinically examined and retrospective data were collected from hospital records.

**Results:**

The women ranged in age from 19.2 to 70.8 years (median 40.8 years) and in height from 103 to 150 cm (median 123 cm). All women had undergone normal pubertal development as assessed by breast development according to Tanner scale and by age of menarche (mean 12.5 yrs., range 11–14 yrs). Despite significant short stature and potentially small pelvic diameters, a well-developed uterus with fairly normal size and shape was found by pelvic ultrasound in most of the patients. Ovarian follicle reserve, assessed by ultrasound was normal in relation to age in all premenopausal women it could be assessed (12 cases). Anti-Müllerian hormone was normal in relation to age in 17 women (89%). HPV was detected in 44% (8/18) and three women carried more than one HPV serotype; findings did not associate with immunological parameters. Three patients had a concurrent cell atypia in Pap smear.

**Conclusions:**

Pubertal development, reproductive hormones and ovarian structure and function were usually normal in women with CHH suggesting fairly normal reproductive health. However, the immunodeficiency characteristic to CHH may predispose the patients to HPV infections. High prevalence of HPV infections detected in this series highlights the importance of careful gynecologic follow up of these patients.

## Background

Cartilage-hair hypoplasia (CHH; MIM #250250) is a rare autosomal recessive metaphyseal chondrodysplasia with an incidence of 1: 23000 births in Finland [1, 2]. It is characterized by severe short-limbed growth failure (mean adult height for males 131 cm and for females 123 cm), thin and sparse hair, and variable immunodeficiency. The risk of malignancies is sevenfold increased, non-Hodgkin lymphoma being the most common malignancy with a 90-fold incidence compared with normal population [3]. CHH is caused by biallelic mutations in the noncoding RNA gene, *RMRP*. *RMRP* mutations disrupt ribosomal processing and cell cycle, leading to defective cell proliferation [4]. CHH is highly variable in severity with phenotypic differences also within families.

While major clinical manifestations of CHH are well-described, almost no data are available on its potential effect on gynecologic and reproductive health and vulnerability to genital infections and HPV-related cancers [2, 5–7]. All these previous studies were based on questionnaires or case reports. None of the previous clinical series included gynecologic examination or laboratory evaluation.

Whether fertility is affected in CHH also remains unclear. In a cohort of 11 adult CHH males, serum concentrations of testosterone, inhibin B and gonadotropins were mainly normal. However, semen analyses showed impaired spermatogenesis [8]. Recent case reports have described two CHH women with hypogonadism [7] and pregnancy outcome of one woman with CHH [6]. There are no other studies evaluating reproduction in patients with CHH.

This lack of data prompted us to carry out a cross-sectional study on gynecologic health in a relatively large cohort of adult women with CHH. We evaluated all subjects clinically and by performing hormonal laboratory measurements and pelvic ultrasound. Pap smear and cervical HPV test were obtained during gynecologic examination.

## Patients and methods

### Patients

**Fig. 1:**
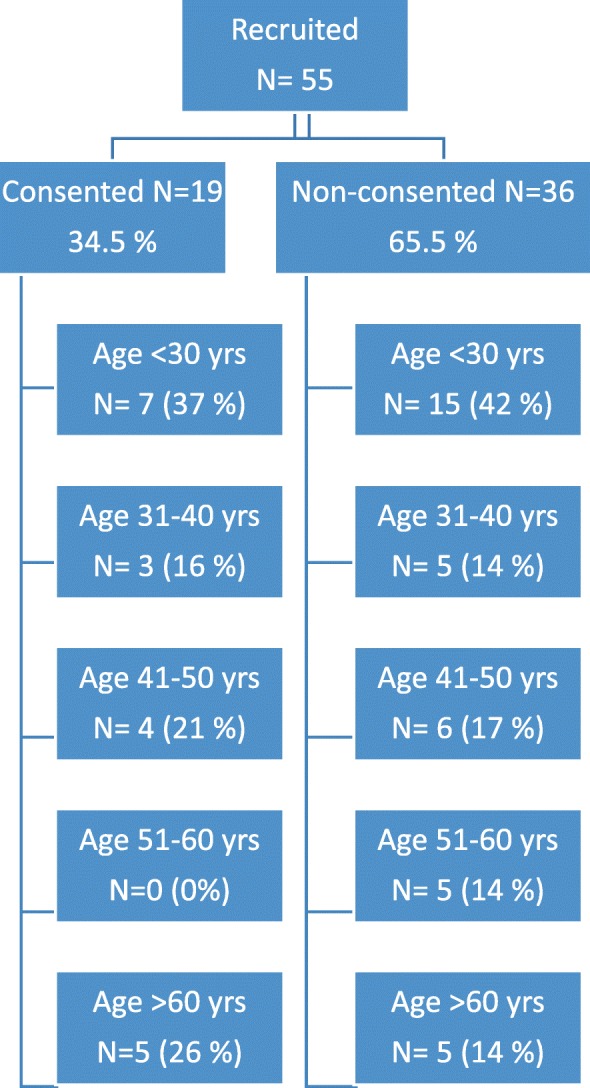
Flow chart showing the proportions and ages of patients who consented and did not consent to the study

The study protocol was approved by the Institutional Review Board of the Children’s Hospital, University of Helsinki and all study participants gave a written informed consent. Patients were identified from the Finnish Skeletal Dysplasia Register which includes > 160 patients with genetically confirmed CHH. All *RMRP* mutations had been detected by Sanger sequencing either at Laboratory HUSLAB, Finland, or as a part of previous or ongoing research at Folkhälsan Institute of Genetics, Helsinki [4, 9]. All 55 women aged over 18 years were invited to the study; 19 CHH females agreed, accounting for a participation rate of 34.5% (Fig.1).

## Methods

Hospital records were reviewed for relevant data. Patients were interviewed for a detailed medical history. Complete gynecologic and pelvic ultrasound examinations (GE Healthcare Voluson S6) were performed by an experienced gynecologist (EH). Pubertal status was recorded according to Tanner’s classification [10]. Antral follicle count (AFC) of the ovaries was considered normal if it was between 10th and 90th percentiles of age-specific normal values [11].

Pap smear and cervical HPV test were obtained when anatomically possible. Cervical samples for HPV test were taken by APTIMA cervical collection and transport KIT and HPV subtypes were analyzed by PCR and Luminex suspension array technology [12]. The test detects 15 high-risk HPV types, six potentially high-risk types and 19 low-risk HPV types.

Blood cell count and serum concentrations of sex hormones, prolactin, thyroid stimulating hormone, gonadotropins and Anti-Müllerian hormone (AMH) were measured during clinical visit. Serum estradiol was extracted from serum with diethyl ether and quantitated by LC-MS/MS (liquid chromatography-tandem mass spectrometry) using a TQ5500 mass spectrometer (AB Sciex, Concord, Canada). The between-run coefficient of variations (CV %) were 4.4–5.1% for serum samples at concentrations 330–900 pmol/L.

Serum luteinizing hormone (LH) and follicle-stimulating hormone (FSH) concentrations were measured using an electrochemiluminescence immunoassay (Abbott Diagnostics, USA). Anti-Müllerian hormone (AMH) was quantitated with an ELISA assay (AMH Gen II ELISA, Beckman Coulter, Brea, CA, USA). Limit of detection (LoD) was 0.08 μg/L and limit of quantitation (LoQ) 0.16 μg/L. Intra-assay and inter-assay CV% was < 6% in the range 3.8–16.5 μg/L. Total CV % was < 8%. Age-specific limits of AMH were applied [13]. The method of AMH measuring was changed in the laboratory during the study. In four patients, Anti-Müllerian hormone (AMH) was quantitated with an electrochemiluminometric assay on a cobase 411 automatic immunoanalyzer (Elecsys AMH Plus, Roche Diagnostics, Mannheim, Germany). Limit of detection (LoD) was 0.01 μg/L and limit of quantitation (LoQ) 0.03 μg/L. Intra-assay CV % was < 2% and inter-assay CV % was < 5% in the range 0.2–19 μg/L of AMH. AMH results with this method are 30% lower than in the ELISA method previously used. For obtaining comparable values by the two different methods, we used mathematical conversion for values detected by Elecsys AMH Plus in four patients (1,3x detected value). For plasma levels of immunoglobulins and lymphocyte counts local laboratory reference values were applied as described previously [14].

### Statistical analysis

Standard statistical methods were used as appropriate. Median, mean and/or standard deviation estimates were computed for variables such as age, length and weight. Fisher’s exact test and logistic regression analysis were applied to examine the correlations of symptoms and laboratory findings.

## Results

### Patient characteristics

The cohort characteristics are described in Table 1 and clinical and laboratory manifestations of immunodeficiency are shown in Table 2. The median age of the 19 study participants was 41.8 years (range 19.2–70.8 yrs). All had undergone a normal spontaneous puberty; the median age at menarche was 12.5 years, varying from 11 to 14 years. Five women were regarded postmenopausal, defined by either i) duration of amenorrhea of more than 12 months and FSH > 30 IU/L (three women) or ii) history of menopausal symptoms and current use of hormonal replacement therapy (two women). Median adult height was 123 cm (103–150 cm) and median weight 41.8 kg (29.0–80.0 kg). Fifteen patients were homozygous for the *RMRP* founder mutation c.70A > G and four patients were compound heterozygous for c.70A > G and c.262G > T mutations.

**Table 1:** Cohort characteristics

	N
Age group (yrs)	
19–30	7
31–40	3
41–50	4
51–60	0
> 60	5
Height (cm)	
< 115	5
115–130	10
> 130	4
Reproductive health	
Menarche (median, yrs)	12.5
Cycle length (variation, median, days)	21–60, 28
Menstrual flow	
sparse	2
normal	15
heavy	2
Pregnancies	6/19
Births	
0	14
1–2	1
> 2	4
Current hormonal therapy	5/19
Combined contraception pills	1
Progestin only -pills	2
Hormone replacement therapy	2
Immunodeficiency	
Suseptibility to infections^a^	
Yes	9
Intermediate	4
Normal	6
Previous skin warts	
Yes	4
No	14
Repeated genital infections	6/19
Previous malignancy^b^	3
Leucocytes (× 10^9^/L) (range, median)	3.2–13.4; 5.6
Lymphocytes (× 10^9^/L) (range, median)	0.5–2.58; 1.22
Neutrophiles (× 10^9^/L) (range, median)	1.74–10.65; 3.37

^a^Yes = one or several episodes of otitis media and/or rhinosinuitits; Intermediate = pneumonia necessitating hospitalization and/or recurrent otitis media and/or rhinosinuitis requiring surgical treatment

^b^1 squamous cell carcinoma,2 basal cell carcinomas

**Table 2:** Clinical and laboratory manifestations of immunodeficiency in 19 patients with cartilage-hair hypoplasia

Pt	HPV	Clinical manifestations of immunodeficiency	Plasma levels of immunoglobulins^a^	Lymphocyte counts^b^	Lymphocyte proliferation^c^	SAD^d^
2	+	NA	NA	NA	NA	NA
3	+	None	Normal	Low TLC, CD3+, CD4+, CD8+, CD19+	NA	NA
11	+	OM, Sin	Normal	Low CD19+	Abnormal	NA
12	+	Sin	Normal	Normal	Abnormal	no
13	+	Sin	NA	NA	Normal	NA
15	+	BE, Pn, Sin requiring surgery	Low IgG	Low CD3+, CD4+, CD19+	NA	NA
16	+	BE, Pn, Sin	Normal	Low CD3+, CD4+, CD8+	Abnormal	yes
17	+	None	Normal	Low TLC, CD8+	NA	NA
1	–	OM, Pn	Normal	Low TLC, CD3+, CD4+, CD8+, CD16/56+	NA	NA
4	–	OM, severe varicella requiring hospitalization	Normal	Low TLC, CD3+, CD4+, CD8+, CD19+	NA	yes
5	–	OM, Sin	Normal	Low TLC, CD3+, CD4+, CD8+	Abnormal	NA
6	–	None	Normal	Low CD3+, CD4+, CD8+, CD19+	Abnormal	NA
8	–	OM	Normal	Normal	Normal	yes
9	–	Sin requiring surgery	Normal	Low CD19+	NA	NA
10	–	OM, Sin requiring surgery	Ig substitution	Low CD3+, CD4+, CD8+, CD19+	Abnormal	yes
14	–	BE, OM, Sin requiring surgery	Normal	Normal	Abnormal	yes
18	–	BE, Pn, Sin requiring surgery	Low IgM	Low CD19+	NA	no
19	–	None	Normal	Normal	NA	NA
7	NA	Boils, OM, autoimmunity	Ig substitution	Low TLC, CD3+, CD4+, CD8+, CD19+	NA	yes

*BE* bronchiectasis, *HPV* human papillomavirus cervical carriage, *Ig* immunoglobulin, *NA* data not available, *OM* recurrent otitis media, *Pn* recurrent pneumonia, *Pt* patient number, *SAD* specific antibody deficiency, *Sin* recurrent rhinosinusitis, *TLC* total lymphocyte count

^a^Local laboratory reference values were applied, measured as described previously in (14)

^b^Local laboratory reference values were applied, measured by flow cytometry as described previously in (14)

^c^Data were obtained from hospital records, measurement performed by various methods

^d^SAD was defined as inadequate antibody response to Pneumovax®: a fourfold rise in antibody titers and post-immunization antibody levels ≥0.35 μg/ml to < 70% of serotypes, measured as described previously in (14)

### Gynecologic status

All patients had normal breast development, either Tanner stage M4 (*n* = 1) or M5 (*n* = 18). Pubic hair was fully developed (Tanner stage P5, *n* = 6) or almost fully developed (P4, *n* = 7) in 68% and partly developed (P3, *n* = 2) or totally absent (P1, *n* = 3) in 32%. Those with P1 had total alopecia.

Pelvic ultrasound examination was performed vaginally in 18 women and abdominally in one woman. In 16 women, the structure of the uterus was considered normal with regard to age, stage of menstrual cycle and the patient’s current hormonal therapy (Table 1). Two postmenopausal patients had small, asymptomatic leiomyomas and one postmenopausal woman had thickened endometrium (4.8–6.3 mm) during continuous hormone replacement therapy.

Median uterine width, determined as the maximum anterior-posterior distance measured in the mid portion of uterine body on a sagittal view, was 27 mm (range 20–38 mm) and median length of uterine corpus, measured from the fundus to the internal orifice of the uterus on a sagittal view, was 38 mm (range 26–70 mm). The 41-year old woman with the smallest, pre-pubertal-size uterus (anterior-posterior measurement 20 mm, corpus length 26 mm) had undergone spontaneous menarche at the age of 11 years, used currently progestin-only pills and had sparse menstrual flow. She had never tried to become pregnant. Another pre-menopausal 46-year old woman also had a relatively small uterus, with corpus anterior-posterior measurement of 20 mm and corpus length of 29 mm. However, she had used levonorgestrel-releasing intrauterine device for years suggesting fairly normal uterine cavity. In all other pre-menopausal women corpus anterior-posterior measurement was > 25 mm and corpus length > 30 mm.

The structure of ovaries could be assessed in 16/19 subjects and all were considered normal in relation to age, phase of menstrual cycle and use of hormonal therapy. In three postmenopausal women (aged 60–70 years) ovaries could not be identified.

AFC in both ovaries could be assessed in 12 premenopausal women and was normal in all. In two cases, AFC from only one ovary could be reliably counted (Table 3).

**Table 3:** Antral follicle count and hormone levels in 14 premenopausal women with cartilage-hair hypoplasia

Study subject	Age group	Current hormonal treatment	Day of menstrual cycle	AFC (N)	FSH (IU/L)	LH (IU/L)	E2 (nmol/L)	AMH (ug/L)
1	19–30	CC	–	18	3.7	2.3	0.179	1.97
2	19–30		9	12	5	3.8	0.643	**0.94**
3	19–30		14	15	14	25.2	0.434	2.29
4	19–30		26	18	5.7	7.2	0.316	4.66
5	19–30	PP	–	7^a^	7.3	4.9	0.07	3.68
6	19–30		14	10	2	0.9	0.264	1.87
7	19–30		20	16	1.8	0.6	0.49	***1.39***
8	31–40		5	14	5.6	4.9	0.32	5.68
9	31–40		17	10	6	12.5	0.579	**< 0.2**
10	31–40		19	3^a^	2.7	1.6	0.423	0.63
11	41–50	PP	–	4	5	1.6	0.081	0.22
12	41–50		20	9	3.1	3.8	0.336	1.34
13	41–50		t.a., am	5	8.5	4.8	0.231	< 0.2
14	41–50		4 mo am	1	68.4	40.9	0.046	< 0.2

*AFC* antral follicle count, *AMH* anti-Müllerian hormone, *CC* combined contraception, *E2* estradiol, *FSH* follicle-stimulating hormone, *LH* luteinizing hormone, *mo* months, *N* number, *PP* progestin-only pill, *am* amenorrhea; *t.a.* endometrium thermoablation done; ^a^Detected from one ovary only. Numbers in **bold** represent values below normal age-specific values and numbers in ***italics*** low normal age-specific values. AMH values detected by two different methods are converted mathematically to correspond each other as described in detail in Methods. Ages are given as age groups to ensure anonymity

### Laboratory parameters

Blood cell count was analyzed in 18/19 cases. Mean hemoglobin was 131 g/L (range 111–146 g/L). Only one woman had hemoglobin under normal range (111 g/L, normal values 117–155 g/l). Mean white blood cell count was 6.3 x e^9^/L (range 3.2–13.4 x e^9^/L) and platelet count 275 x e^9^/L (range 147–398 x e^9^/L).

AMH was measured from all 19 patients and was under detection limit in all postmenopausal women (*n* = 5) and in the two perimenopausal women (aged > 45 yrs). Most of the premenopausal women had normal AMH according to age-specific reference values (Table 3). Two women had lower than expected AMH levels and there was a discrepancy between the age-specific AMH level and AFC detected by vaginal ultrasound. The first one (patient #2, Table 3) was a young woman with normal AFC (12) while AMH of 0.94 μg/L suggested lower ovarian reserve. The other patient (patient # 9, Table 3) had normal AFC (10), but AMH was under detection level (< 0.2 μg/L). Both had regular cycles (period 28) and did not use hormonal therapy. One patient had low-normal level of AMH with normal AFC (patient # 7, Table 3). In none of the patients both AFC and AMH suggested lower than normal age-specific ovarian reserve.

FSH, LH and estradiol levels were consistent with the phase of cycle or use of hormone treatment (Table 3). Thyroid stimulating hormone and testosterone levels were all in normal range.

Prolactin level was increased in two women (2460 mU/L and 588 mU/L; normal range 50–500 mU/L), both women were on psychiatric medication affecting prolactin secretion.

### Pap smear and HPV-tests

Pap smear and cervical HPV test were obtained from 18/19 cases. Altogether 9 women had abnormal findings; these are presented in Table 4. Eight of the 18 women (44%) had a positive HPV test. Five were positive for one subtype, three of which were of high-risk types. Another two women had two high-risk subtypes. Finally, one woman tested positive for three different HPV subtypes, one of which was classified as a high-risk type. We found no correlation between the HPV status and any other clinical manifestations of immunodeficiency or previously measured blood lymphocyte subset counts or serum immunoglobulin levels (Table 2). All patients with high-risk HPV had recently undergone thorough immunologic evaluation with normal counts of T and B cells and normal immunoglobulin A, M and G levels.

**Table 4:** Abnormal Pap smear and human papilloma virus test results in nine women with cartilage-hair hypoplasia. Ages are given as age groups to ensure anonymity

Study subject	Age group	Previous abnormal Pap smear	Current Pap smear	Positive HPV test	HPV types	High-risk HPV type
1	19–30	NA	ASC-US	–	–	–
2	19–30	NA	ASC-US	+	42	No
3	19–30	Yes	LSIL	+	56,(66,67)	Yes
11	41–50	Yes	Normal	+	16	Yes
12	41–50	Yes	ASC-US	+	52,59	Yes
13	41–50	No	Normal	+	56	Yes
15	> 60	No	Normal	+	30	No
16	> 60	No	Normal	+	74	No
17	> 60	No	Normal	+	16,18	Yes

*ASC-US* atypical squamous cells of undetermined significance, *HPV* human papilloma virus, *yrs* years, *LSIL* low-grade squamous intraepithelial lesions, *NA* data not available

## Discussion

Little is known about gynecologic health in women with CHH and our study provides important novel data that can be implemented in the management and follow-up of patients with CHH and increase our knowledge on gynecological issues more widely among women with skeletal dysplasia. The main findings of our study include normal pubertal development, mostly normal genital anatomy and a high prevalence of HPV positivity.

We have previously described two young CHH women with absent spontaneous pubertal development and reduced ovarian reserve [7]. However, in this study consisting of 19 women with CHH and severe disproportional growth failure, pubertal development and sexual maturation were normal as assessed by Tanner scale and age of menarche. Further, the size of uterus was within normal range in 18/19 women. In one woman, at age 41 years, the size of uterus resembled pre-pubertal uterus [15, 16]. However, she had a spontaneous menarche and history of regular cycles suggesting fairly normal estrogen production. It remains unknown whether the small uterus is due to direct effects of aberrant *RMRP* function or mediated by e.g. endocrine factors. AFC was normal in relation to age in all those who could be evaluated, suggesting fairly normal fertility potential in this patient group. The lack of pubarche in three patients is likely to be a result from impaired germ-cell proliferation in the hair follicles.

For practical reasons blood tests for hormonal measurements were taken during clinical appointment. Three women were on hormonal contraception at that time. Combined contraception may decrease the level of AMH and number of AFC resulting in underestimation of fertility potential [17, 18]. However, in a woman using combined contraception in our series, AMH and AFC were normal. In two other patients, progestin only pills were used. Their effect on hormonal levels is less evident than that of combined contraception [17].

Sexually active CHH women are at risk for acquiring HPV and combined immunodeficiency may predispose them to prolonged or severe infection. A Finnish series of women attending HPV screening during 2003–2005 showed age –specific HPV positivity to vary from 2.8–24.2% in women aged 25–65 years [19]. In our series one or more HPV subtypes were found in 44% (8/18) patients with CHH. Two of our patients with HPV were under 25 years of age, and the prevalence in this age-group is known to be higher [20]. However, spontaneous recovery of HPV infection is more common in the young age group. Five of those with HPV (62.5%, 5/8) had high-risk HPV subtypes. In the Finnish population, high-risk subtypes have been found in 41.2–65.3% of HPV positive screening samples [19]. Since prolonged infections with high-risk HPV subtypes predispose to cervical cancer, careful and systematic follow-up of abnormal Pap smears is of utmost importance in immune deficient CHH patients. Also, HPV vaccination should be considered. In our patients with high-risk HPV carriage, normal lymphocyte subsets and immunoglobulin levels have been documented previously, highlighting the importance of screening also CHH patients with subclinical immunodeficiency.

Most of the CHH women in our series were or had been sexually active, indicating a need for reliable and suitable contraception. In a previous study, intrauterine device (IUD) was used only by 8% of short-statured women [5]. This may be explained by fear of potential difficulties or complications in IUD insertion caused by disproportionate pelvic diameters and the fact that Copper IUD can exacerbate menstrual cramps and menstrual flow. However, nowadays, levonorgestrel releasing IUD is an effective contraceptive method treating also heavy menstruation. Based on the possibility of performing clinical gynecologic examination and the normal uterine measurements found in the present study, we regard the use of levonorgestrel releasing IUD a valuable option for women with CHH. By reducing menstrual flow, it may also have a positive effect on potential anemia due to bone marrow failure, a possible complication in CHH.

Our study is limited due to the relatively small size of the cohort and the low participation rate which may cause bias. However, in rare diseases larger cohorts are often impossible to obtain. In CHH, severe short stature further complicates participation of individuals living far from the study center. The participation rate was comparable to our previous studies on CHH. Further, our study permit did not allow us to collect data on the offspring’s health, including postnatal growth. Despite these limitations our study is the largest gynecological evaluation ever performed in CHH or any other skeletal dysplasia and thus provides valuable new data. Further studies are needed to further elucidate the largely unknown field of gynecological health in women with skeletal dysplasia and the outcomes of their pregnancies.

## Conclusions

Immunodeficiency and increased risk for malignancies contribute to significant morbidity in CHH patients. While major clinical manifestations of CHH are well-described, only little is known about gynecologic health in CHH. In this prospective series reproductive hormones and health were fairly normal. However, high prevalence of HPV infections detected in this series highlights the importance of careful gynecologic follow up of these patients since immunodeficiency may predispose CHH patients to HPV infections.

## Abbreviations

AFC: Antral follicle count
AMH: Anti-Müllerian hormone
CHH: Cartilage-hair hypoplasia
CV: Coefficient of variation
FSH: Follicle-stimulating hormone
HPV: Human papillomavirus
LH: Luteinizing hormone
LoD: Limit of detection
LoQ: Limit of quantification
